# Ductal carcinoma in situ of the male breast: clinical radiological features and management in a cancer referral center

**DOI:** 10.1007/s10549-022-06689-y

**Published:** 2022-09-17

**Authors:** Luca Nicosia, Germana Lissidini, Manuela Sargenti, Anna Carla Bozzini, Gabriel Farante, José Vila, Chiara Oriecuia, Eleonora Pagan, Vincenzo Bagnardi, Matteo Lazzeroni, Filippo Pesapane, Claudia Sangalli, Viviana Galimberti, Enrico Cassano, Paolo Veronesi

**Affiliations:** 1 Department of Breast Radiology, European Institute of Oncology (IEO), IRCCS, 20141 Milan, Italy; 2 Division of Breast Surgery, European Institute of Oncology (IEO), IRCCS, Milan, Italy; 3 Hospital Universitario y Politécnico La Fe, Valencia, Spain; 4 Department of Clinical and Experimental Sciences, University of Brescia, Brescia, Italy; 5 Department of Molecular and Translational Medicine, University of Brescia, Brescia, Italy; 6 Department of Statistics and Quantitative Methods, University of Milan-Bicocca, Milan, Italy; 7 Division of Cancer Prevention and Genetics, European Institute of Oncology (IEO), IRCCS, Milan, Italy; 8 Data Management, European Institute of Oncology IRCCS, 20141 Milan, Italy; 9 Department of Oncology and Hemato-Oncology, University of Milan, 20133 Milan, Italy

**Keywords:** Male DCIS, Breast, Disease-free survival, Surgery

## Abstract

**Purpose:**

To present an overview of the management of male patients with Ductal Carcinoma In Situ of the breast (male DCIS).

**Methods:**

We retrospectively studied all male patients with a diagnosis of pure DCIS from January 1999 to December 2018: 20 patients were identified in our cancer referral center. We collected data regarding clinical presentation, age of onset, radiological features, receptor status of the neoplasm, histological type, and the follow-up of those patients.

**Results:**

The median age was 62 years (range 21–80). All patients underwent surgery, in 15/20 (75%) cases a mastectomy was carried out. Two patients (10%) underwent endocrine treatment and 1/20 (5%) underwent radiotherapy. The receptor status for 15/20 patients was documented: 13/15 patients were ER+/Pr+. In 3 cases the Ki 67% was positive (i.e., > 20%). All cases were negative for Her2. The median follow-up time was 9.0 years (IQR 4.0–13.7). Only one patient had an ipsilateral recurrence with the finding of an infiltrating carcinoma in the same breast after 14 years. The 5-year disease-free survival was 92.9%.

**Conclusion:**

Pure DCIS in men is an extremely rare disease: proper diagnosis and management allow an excellent prognosis.

## Introduction

Breast cancer in women is still, nowadays, one of the most dangerous and frequent malignancies, accounting for 12% of all new annual cancer cases worldwide [1, 2]. Conversely, male breast cancer is a rare disease and comprises only about 1% of all male malignancies, with an annual incidence in Europe of around 1/100,000 men [3, 4].

Ductal carcinoma in situ (DCIS) of the breast is defined as a lesion confined to the breast ducts, without invasive features or metastatic potential [5, 6]. Pure DCIS represents approximately 10% of all male breast cancers and less than 0.1% of all types of cancers in men [7].

The early diagnosis of male carcinoma in situ and adequate clinical therapeutic management is essential to avoid the evolution to a worse type of disease (e.g., infiltrating carcinoma).

Considering the rarity of this disease, little data is available and very few case studies have been published [7, 8]: any work presenting diagnostic, clinical, and therapeutic options can be valuable for the adequate management of those patients.

The aim of this paper is to present an overview of our patient management: the most common clinical and radiological manifestations, the most common receptor status. We also want to present follow-up data over a long period of time. Greater awareness of this rare disease, with potentially important implications, can help in standardizing the proper management of this type of patient.

## Methods

**Fig. 1:**
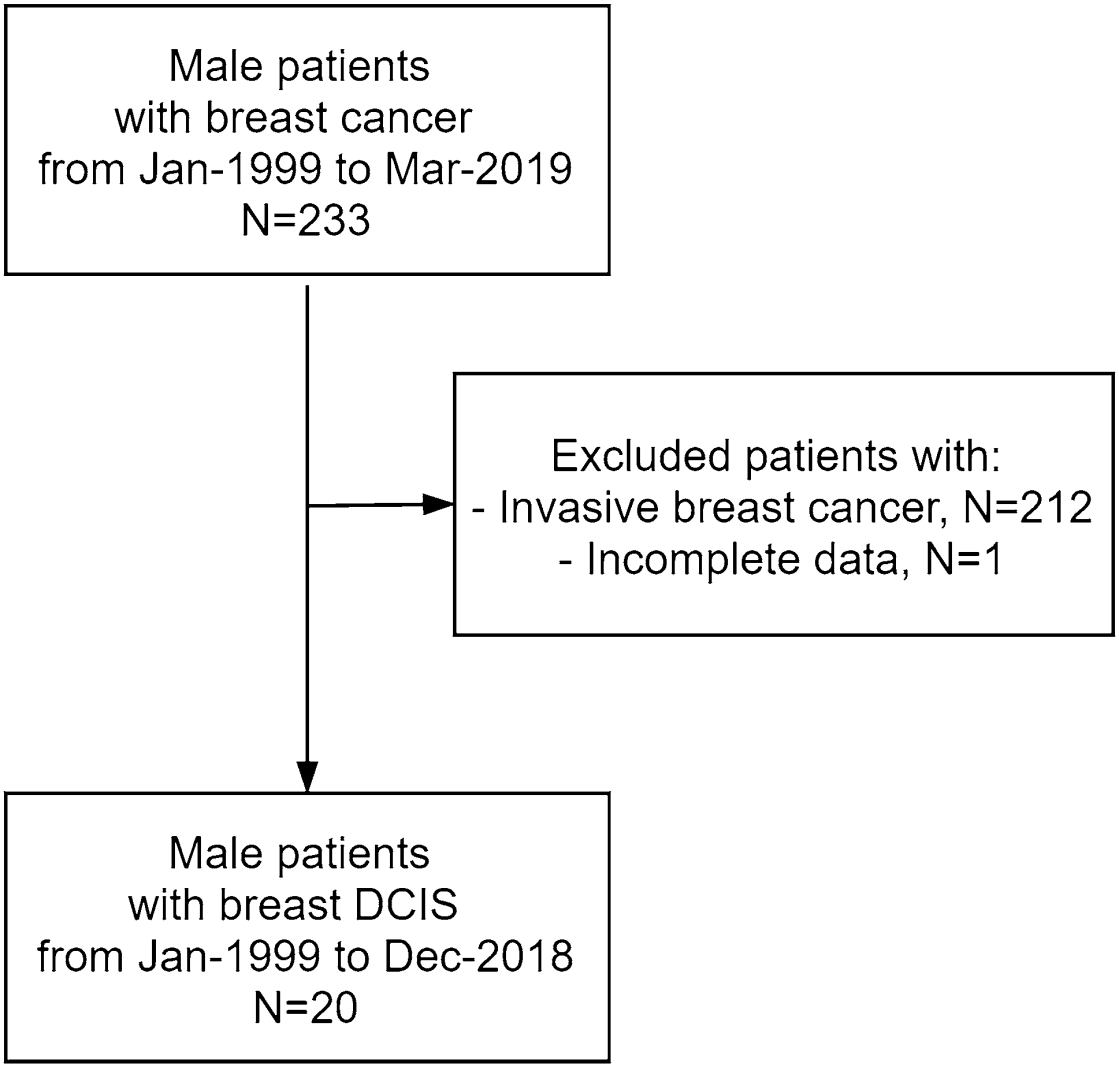
Flow chart of the study’s inclusion and exclusion criteria

This retrospective study was registered with the Ethics Committee and was approved by the Institutional Review Board. We retrospectively studied all male patients with a diagnosis of pure DCIS from January 1999 to December 2018: 20 patients were identified in our cancer referral center (European Institute of Oncology Milano). We included in the study all male patients with a histological diagnosis (at surgery) of pure breast DCIS. We collected data regarding the clinical presentation, radiological presentation, age of onset, histological type, receptor status of the neoplasm, treatment and the follow-up of those patients. We excluded patients without a pure breast DCIS (for example with infiltrating components), and patients who were not operated or who did not have complete follow-up data. A flow chart of the study’s inclusion and exclusion criteria is shown in Fig. 1.

### Statistical analysis

Continuous variables were shown as means or medians with interquartile ranges (IQR) or min–max ranges, dichotomous variables as counts and percentages.

Endpoints evaluated were disease-free survival (DFS) and overall survival (OS). DFS was defined as the time from surgery until local recurrence, metastasis, other primary carcinomas, or death, whichever occurred first. OS was defined as the time from surgery until death (from any cause). The OS and DFS functions were estimated with the Kaplan–Meier method.

Statistical analyses were performed using SAS statistical software version 9.4 (SAS Institute, Inc., Cary, NC, USA).

## Results

On a series of 233 patients with male breast cancer operated in our institute, pure DCIS was observed in 21 cases (9.0%). One patient was excluded due to a lack of follow-up data (Fig. 1).

The median age was 62 years (range 21–80), and 2 (10%) were younger than 40 years.

### Clinical presentation

Clinical gynecomastia was present in 5/20 (25%) cases. Bloody nipple discharge was present in 9/20 cases (45%). A clinically palpable mass was present in 11/20 (55%) cases (Table 1).

**Table 1:** History and clinical manifestation

	Overall *N* = 20 N (%)
Age	
< 40 years	2 (10)
≥ 40 years	18 (90)
Median (IQR/min–max)	62 (46–67/21–80)
With family history of breast cancer	5 (25)
With gynecomastia	5 (25)
Bloody nipple and/or palpable mass	
None	2 (10)
Only bloody nipple	7 (35)
Only palpable mass	9 (45)
Both bloody nipple and palpable mass	2 (10)
Preoperative diagnosis	
Citology (nodule and/or secretion)	12 (60)
Resection	5 (25)
Unknown	3 (15)

### Radiological presentation

In 10/20 cases preoperative radiological examinations were available. In particular: 3/20 patients performed only mammography; 4/20 cases performed both mammography and breast ultrasound; 3/20 patients performed only breast ultrasound. In 3/10 cases the presentation was a well-defined nodule (all of them with a cystic component). In 5/10 cases the presentation was a poorly defined nodule (1 of them with a cystic component). In 2/10 cases, no mammographic findings were seen in the presence of bloody nipple discharge only.

**Fig. 2:**
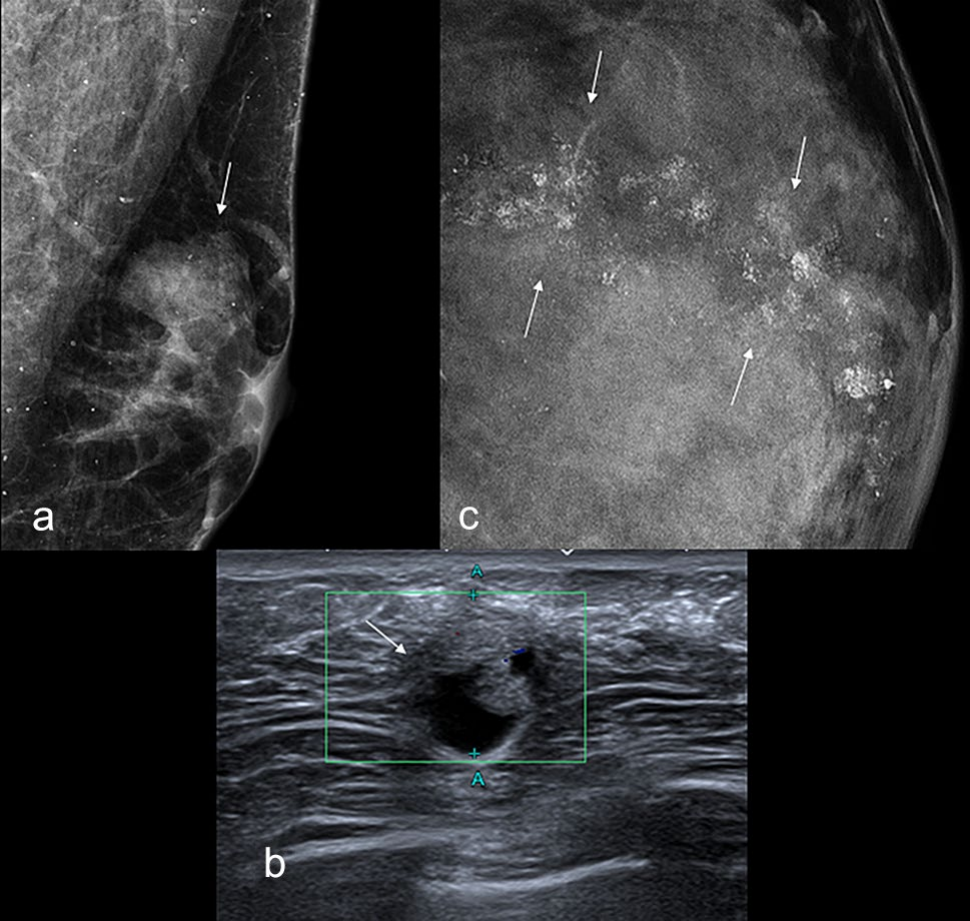
**a** Case of intermediate-grade in situ breast neoplasm. A 53-year-old man presented to our attention for the finding of a palpable mass of the left breast associated with nipple bleeding. In Fig. 2a we can see the typical mammographic presentation: a nodule with well-defined margins without significant associated microcalcifications (arrow). The typical ultrasound presentation is presented in **b**. A nodule with well-defined margins, with a cystic component, lacking vascularization on color doppler (arrow). Based on our experience, this type of mammographic and ultrasonographic presentation always requires further cytological/histological investigation. In contrast, **c** presents the classic presentation of carcinoma in situ in women. In this case, the 45-year-old woman presented to our attention for a screening mammogram in the absence of clinical findings. The mammogram shows a series of extensive suspicious polymorphic microcalcifications (arrows): one of the typical manifestations of DCIS in women.

Only two cases (5%) showed microcalcifications with a poorly defined nodule. Unfortunately, in 10 cases the preoperative diagnostic examinations were performed at another institution, and the type of examination or its diagnostic images could not be retrieved. Radiological features are summarized in Table 2. Some examples of typical radiological presentations are shown in Fig. 2 a–c.

**Table 2:** Radiological features

	Overall *N* = 20 N (%)
Type of preoperative radiological examinations	
None	10 (50)
Only mammography	3 (15)
Only breast ultrasound	3 (15)
Both mammography and breast ultrasound	4 (20)
Radiological findings and cystic component	(n=10)
None	2 (20)
Well-defined nodule w/o cystic component	0
Well-defined nodule w cystic component	3 (30)
Poorly defined nodule w/o cystic component	4 (40)
Poorly defined nodule w cystic component	1 (10)
Dimensions (millimeters)	(n=8)
Mean, min–max	19.3 (5–25)
Patients with missing information	1
With microcalcifications	2 (5)

*w/o*, without; *w*, with

#### Type of preoperative diagnosis

In 12/20 cases a cytological examination was performed (or on nodule or on blood secretion). In the other cases with available documentation (5 cases), a direct breast diagnostic resection was performed following a doubtful clinical radiological finding (Table 1).

### Surgical features and management

In 15/20 cases the patients were treated with mastectomy, in 5/20 cases with lumpectomy. Sentinel lymph node biopsy was performed in 13 of 20 cases. In no case were metastatic lymph nodes evident. None of the patients underwent axillary dissection. Surgical features and management are shown in Table 3.

**Table 3:** Surgical features and management

	Overall *N* = 20 N (%)
Type of surgery	
Mastectomy w/o SLNB	3 (15)
Mastectomy w SLNB	12 (60)
Lumpectomy w/o SLNB	4 (20)
Lumpectomy w SLNB	1 (5)
Number of SLN removed	(n=13)
Mean, min–max	1.8 (1–6)
Number of positive SLN	(n=13)
Mean, min–max	0
Axillary dissection	0
Systemic neoadjuvant treatment	
No	20 (100)
Yes	0
Endocrine therapy	
No	18 (90)
Yes	2 (10)
Radiotherapy	
No	19 (95)
Yes	1 (5)

*w/o* without, *w* with, *SLNB* sentinel lymph node biopsy

### Histological features

We have the receptor status for 15/20 patients. In 13/15 cases the patients were ER+/Pr+; one patient was Er+/Pr−; one patient was Er−/Pr−. Ki 67 was considered positive if ≥ 20% [9]. Ki 67 was positive in only 3 cases.

In 14/15 cases Her2 was negative while for 1 patient the HER2 status was unknown. Most of the cases were papillary subtypes and all the cases were low/intermediate DCIS.

Specifically, in 3/20 cases we had low-grade papillary and cribriform DCIS; in 1/20 cases we had low-grade pure papillary DCIS. In 1/20 cases we had low-grade pure cribriform DCIS. In 4/20 cases we had intermediate-grade papillary and cribriform DCIS. In 2/20 cases we had intermediate-grade pure cribriform DCIS. In 4/20 cases, we had intermediate-grade pure papillary DCIS. In 5/20 cases, we had intracystic papillary carcinoma. Histological features are shown in Table 4.

**Table 4:** Histological features

	Overall *N* = 20 N (%)
Histological type	
Intracystic papillary carcinoma	4 (20)
Low-grade DCIS	5 (25)
Intermediate-grade DCIS	11 (55)
ER/PR	
ER+/PR+	13 (65)
ER+/PR−	1 (5)
ER−/PR−	1 (5)
Unknown	5 (25)
Ki67 (%)	
< 20%	12 (60)
≥ 20% (positive)	3 (15)
Unknown	5 (25)
HER2 status	
Negative	14 (70)
Positive	0 (0)
Unknown	6 (30)
Histological subtype	
Low-grade papillary and cribriform DCIS	3 (15)
Low-grade pure papillary DCIS	1 (5)
Low-grade pure cribriform DCIS	1 (5)
Intermediate-grade papillary and cribriform DCIS	4 (20)
Intermediate-grade pure papillary DCIS	4 (20)
Intermediate-grade pure cribriform DCIS	2 (10)
Intracystic papillary carcinoma	5 (25)

### Treatment and follow-up

Most patients did not undergo treatment after surgery. Only two patients (10%) underwent endocrine treatment and 1/20 (5%) underwent radiotherapy.

The median follow-up time was 9.0 years (IQR 4.0–13.7 years). Only one patient had an ipsilateral recurrence with the finding of infiltrating carcinoma at the same breast after 14 years. The first surgery for this patient was a lumpectomy.

**Fig. 3:**
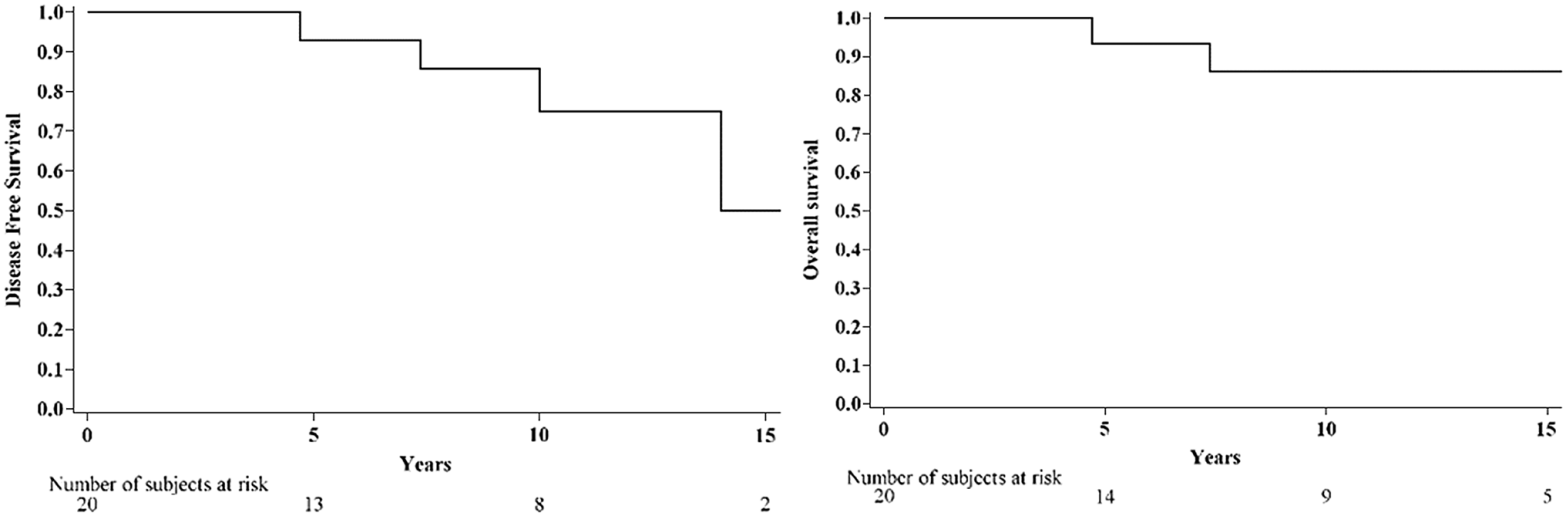
Kaplan–Meier curves for disease-free survival and overall survival

One patient reported a second primary prostate cancer after 10.0 years and two patients died after 4.7 and 7.4 years, respectively, for causes unrelated to breast cancer. The 5-year DFS was 92.9% (95% CI 59.1–99.0) (Table 5). The overall survival and disease-free survival Kaplan–Meier curves are shown in Fig. 3.

**Table 5:** Disease-free survival and overall survival

	*N* = 20
Median time of follow-up [years] (IQR)	9.0(4.0−13.7)
Disease-free survival (DFS)	
Observed events, N (%)	4 (20%)
Loco-regional events, N	1^a^
Distant events, N	0
Other events, N	3^b^
5-year DFS (95% CI)	92.9 (59.1–99.0)
10-year DFS (95% CI)	85.7 (53.9–96.2)
Overall survival (OS)	
Observed deaths, N (%)	2 (10%)
Other/Unknown causes, N	2
5-year OS (95% CI)	93.3 (61.3–99.0)
10-year OS (95% CI)	86.2 (55.0–96.4)

^a^Patient with ipsilateral recurrence with the finding of infiltrating carcinoma diagnosed after 14.0 years

^b^One patient reported a second primary prostate cancer after 10.0 years and two patients died after 4.7 and 7.4 years, respectively

## Discussion

Pure ductal carcinoma in situ (DCIS) of the male breast is a very rare disease with few cases described in the literature, mainly case series or case reports [4, 7, 8, 10, 11, 12, 13, 14, 15, 16]. In fact, research on this type of pathology and clinical evidence are limited. In this article, based on our experience, we aim to provide an appropriate diagnostic and therapeutic approach for this rare condition. Radiological, histological, and clinical features in male DCIS differ from DCIS in women, and for adequate management, the knowledge of two different forms of the same pathology appears extremely important [17, 18, 19, 20, 21]. In our series, pure DCIS was observed in 21/233 cases (9.0%). Data are in line with those from the Surveillance, Epidemiology, and End Results (SEER) database of the National Cancer Institute [22]: male in situ carcinoma was observed in 280 of 2984 male breast cancer cases (9.4%) diagnosed between 1973 and 2001. According to the SEER data [22], diagnosis of male DCIS occurs at an older age compared to women: 62 years compared to 58 years. Our research confirms this analysis with a median age at diagnosis of 62 (range 21–80 years). In 5 cases (25%), a family history of breast neoplasia was reported.

The presentation of this type of pathology is different from that of women: in particular, in women, DCIS is often clinically occult and occurs, mostly, in the form of microcalcifications [23, 24]. In our series of male DCIS, microcalcifications were evident in only two cases. In our experience, the carcinoma in situ presented itself as a palpable nodule (in 55% of cases). Most of our cases were low/intermediate-grade ductal carcinoma in situ and in the 25% of our cases, the main histological was the intracystic papillary carcinoma: the male breast is typically composed of a nipple with large central ducts, mostly of the papillary type [8]. Furthermore, in 10% of cases, the radiological manifestation was that of a predominantly cystic nodule. From our experience, nipple blood discharge should always be investigated with cyto/histological examination even without radiological findings. Considering the low breast thickness, cytology was often preferred to breast biopsy although it obviously provided less pre-operative information. Also, in our experience, cytological evaluation of male breast lesions provides excellent diagnostic performance [25].

In summary, based on our experience, we could make the following suggestions for the management of patients with male breast DCIS.

- Patients with high familiarity for breast neoplasms and a BRCA mutation should undergo a breast examination and breast ultrasound once a year [26].
- Nipple blood secretion should always be investigated by cytological examination of the secretion (even in the absence of associated suspicious breast radiological findings).
- Gynecomastia should always be investigated with at least one ultrasound examination in order to decide a possible cyto-microhistological sampling.
- Any breast lump, even with a predominantly cystic component, should always be investigated by micro histological examination.
- The therapeutic treatment of choice (also to reduce the rate of recurrence) should be mastectomy with associated sentinel lymph node biopsy: it is estimated that up to 26% of patients with a preoperative diagnosis of DCIS are upgraded to invasive carcinoma on final postoperative histological examination [27]. In view of this axillary evaluation, including SLNB, could be justified in male DCIS patients undergoing mastectomy because of the possibility of upstaging to invasive cancer at surgery: vacuum-assisted biopsy (especially with macroscopic removal of the lesion) is the best way to decrease biopsy underestimation in breast DCIS [17], anyway the poor of the breast thickness makes the vacuum-assisted breast biopsy scarcely used in males. If the decision is made not to use sentinel lymph node biopsy in the male with DCIS, an extemporaneous intraoperative diagnostic examination of the surgical piece is suggested to confirm the in situ nature of the neoplasm.
- Endocrine and/or radiation treatment is not commonly suggested in male patients with DCIS, although it has already been explored in the literature [28]. However, according to our experience, it can be considered, after multidisciplinary discussion, in cases that might have a worse prognosis (such as cases with intralesional necrosis or high ki 67 values).

In most of our cases, the patients were positive for estrogen and progesterone receptors and had low ki67 (< 20%). Prognosis of patients with carcinoma in situ of the male breast is excellent with 5-year overall survival of 93.3%. These data are quite similar to the survival data for DCIS in women [29]. A prompt diagnosis is crucial to avoid any evolution towards a more aggressive form of the disease.

The main limitation of this study is its retrospective nature so some data of interest in some of our patients could not be retrieved. It would be advisable a multicentric and prospective study based on common registration criteria and management to obtain additional critical information.

## Conclusion

Pure DCIS in men is an extremely rare disease. Knowledge of appropriate management is therefore limited and not very standardized. In this article, we present the main features of our case series in a cancer referral center. Early recognition of this pathology and proper management will allow the best treatment options and an excellent prognosis for these patients.

## Data Availability

The datasets used and/or analyzed during the current study are available from the corresponding author on reasonable request.
